# Natural products as therapeutics for malignant melanoma: preclinical evidence and mechanism 

**DOI:** 10.3389/fphar.2025.1641838

**Published:** 2025-08-26

**Authors:** Hongjin Gao, Jianli Huang, Dengfeng Zhang, Sixuan Li, Shengzhi Long, Yuesi Qin

**Affiliations:** ^1^ Chengdu Integrated TCM & Western Medicine Hospital, Chengdu, Sichuan, China; ^2^ Dermatology Department of Bijie Traditional Chinese Medicine Hospital, Guiyang, Guizhou, China

**Keywords:** melanoma, natural products, signaling pathway, apoptosis, biological activity

## Abstract

Melanoma is one of the most common malignancies among fair-skinned populations. Natural products, a diverse group of bioactive compounds derived from plants and animals, have demonstrated inhibitory effects on melanoma growth, invasion, and metastasis. This review summarizes the mechanisms through which natural products inhibit melanoma progression and metastasis. These compounds are categorized based on their mechanisms of action. Many natural products have been found to induce apoptosis in melanoma cells through various signaling pathways. For instance, rhodopsin and the triazolylpeptidyl penicillin derivative TAP7f suppress the Wnt/β-catenin signaling pathway, thereby reducing melanoma cell proliferation and migration. Resveratrol and vitamin E delta-tocotrienol (δ-TT) inhibit caspase-dependent mitochondrial and endoplasmic reticulum stress pathways, inducing apoptosis in melanoma cells. Shikonin and plumbagin exert their antitumor effects through the mitogen-activated protein kinase/extracellular signal-regulated kinase (MAPK/ERK) signaling pathway. In addition, natural products such as silymarin, capsaicin, and ursolic acid exhibit multi-targeted anticancer effects with high efficiency and low toxicity by modulating various signaling pathways. These findings highlight the ability of natural compounds to regulate multiple biological targets, offering new directions and potential clinical applications in melanoma therapy. Natural product–based drug development holds great promise for overcoming current limitations in cancer treatment.

## 1 Introduction

Random accumulation of cellular mutations or genetic defects can transform normal melanocytes into malignant melanoma. Although malignant melanoma accounts for less than 5% of all skin cancers, it is responsible for the majority of skin cancer–related deaths (Wilczak et al., 2025). The incidence of malignant melanoma varies by population, based on the current incidence rate, it is estimated that by 2040, the number of new melanoma cases will increase by 50%. According to 2020 data, the melanoma-related mortality rate is projected to rise by 68%, with the highest incidence observed in white populations (Long et al., 2023; Connor et al., 2025). Cutaneous malignant melanoma is growing faster than any other cancer and thus poses a significant health threat worldwide.

**FIGURE 1:**
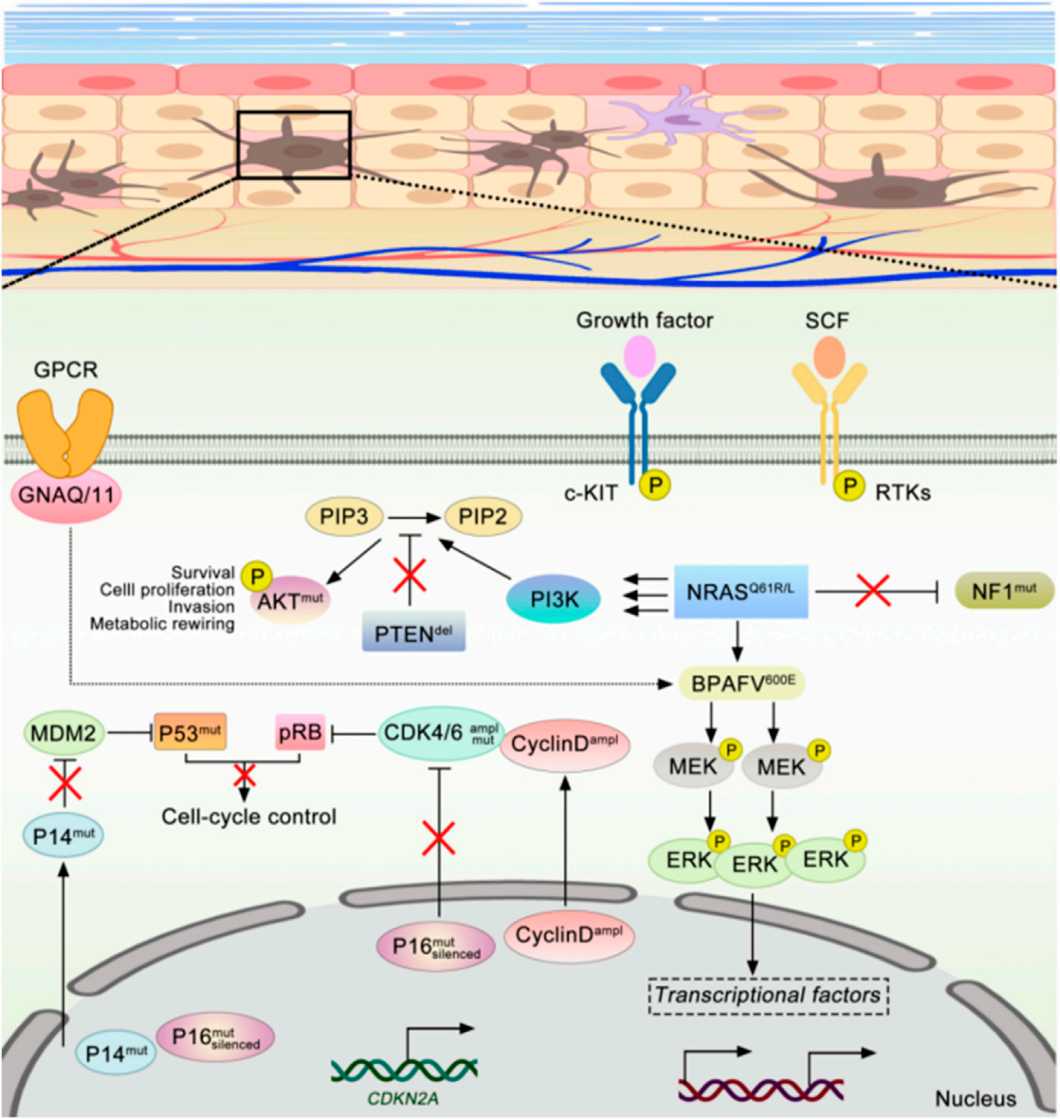
The occurrence and development of melanoma are closely related to multiple signaling pathway mechanisms.

Most melanomas arise from somatic mutations that are acquired later in life. Several recently known key genetic mutations in melanoma, such as BRAF, NRAS, PTEN, MITF, CDKN2A, KIT, and TP53, have been described as crucial factors in melanoma development (Zhang et al., 2016; Palmieri et al., 2018; Castellani et al., 2023). Approximately 50% of malignant melanomas have mutations in BRAF, which promote melanin production through conformational activation of the MAPK (RAS/RAF/MEK/ERK) signaling pathway. This mediates several phenomena, including cell proliferation, differentiation, and secretion of signaling molecules associated with the appearance and progression of melanoma (Czarnecka et al., 2020; Ottaviano et al., 2021). Mitogen-activated protein kinase (MAPK) signaling pathway is one of the melanoma’s most common mutational pathways. It was estimated that 70% of melanomas contained mutations in the MAPK signaling pathway (Reddy et al., 2017; Cheng et al., 2018; Davis et al., 2019), and 50% of melanomas contained activated BRAF (mostly V600E) mutations (Scolyer et al., 2011), another 14%–20% of melanomas had NRAS mutations (Yakovian et al., 2021), and 2% had CKIT mutations (Kim and Kim, 2024), as shown in Figure 1.

Surgical resection remains the preferred treatment for melanoma diagnosed at an early stage, before metastasis has occurred. However, once metastasis occurs, alternative therapeutic strategies are required. Chemotherapy has traditionally been used to alleviate symptoms, control tumor progression, or, in rare cases, achieve remission. It relies on cytotoxic agents to inhibit the abnormal proliferation of cancer cells or slow their overall growth rate (Long et al., 2023). Metastatic melanoma chemotherapy drugs include dacarbazine, paclitaxel, platinum compounds, and temozolomide (Megahed and Koon, 2014). Therapies can counteract the molecular defects present in melanoma, among which the most effective are BRAF inhibitors, which are used to treat metastatic and unresectable BRAF-mutated melanoma (Pelosi et al., 2024). It is highly effective in about half of patients with BRAF-mutated melanoma. Immersive checkpoint inhibitors are the most effective drugs for metastatic melanoma (Byrne and Fisher, 2017). Currently, the anti-CTLA-4 antibody (ipilimumab) and two anti-programmed cell death (PD-1) (nivolumab and pembrolizumab) have been used in clinical (Rotte et al., 2015). Although the immune checkpoint inhibitors are therapeutically effective, inevitable complications inhibit the mechanisms promoting self-cellular tolerance (Sharma and Allison, 2015). With the emergence of targeted therapy and immunotherapy over the past decade, significant progress has been made in the treatment of melanoma. However, ongoing challenges—such as limited therapeutic efficacy and disease recurrence—continue to drive the development of novel approaches and combination strategies.

Natural products and their derivatives are characterized by diverse structures, biological activities, low toxicity, and comprehensive sources. Their role in developing new anti-cancer drugs and lead drug compounds is becoming increasingly important (Agarwal et al., 2020; Kang et al., 2025; Wang et al., 2025). In addition, over the past 2 decades, many dietary and natural compounds with physiological activity—including phenols, flavonoids, alkaloids, carotenoids, gingerols, and organosulfur compounds—have been shown to inhibit both early and late stages of cancer. As a result, increasing attention has been directed toward the use of novel natural compounds in melanoma treatment, which has become an active area of research globally. For example, ginsenoside Rg3 and topotecan (a derivative of camptothecin) have already been approved for clinical use. In contrast, compounds such as luteic acid and silybin are still undergoing clinical evaluation. Their anticancer effects involve autophagy, apoptosis, and the regulation of multiple signaling pathways (Wen et al., 2021). This review focuses on natural products in the treatment of malignant melanoma and highlights their underlying molecular mechanisms. The identification of novel antitumor agents from natural products represents a promising strategy to improve long-term survival in melanoma patients and serves as a valuable source for anticancer drug discovery. Natural antitumor agents act through diverse mechanisms to inhibit cancer progression, including suppression of malignant cell proliferation, invasiveness, and neoangiogenesis, while typically exhibiting lower toxicity than conventional chemotherapeutic agents.

## 2 Natural products targeting melanoma-related signal pathways

**FIGURE 2:**
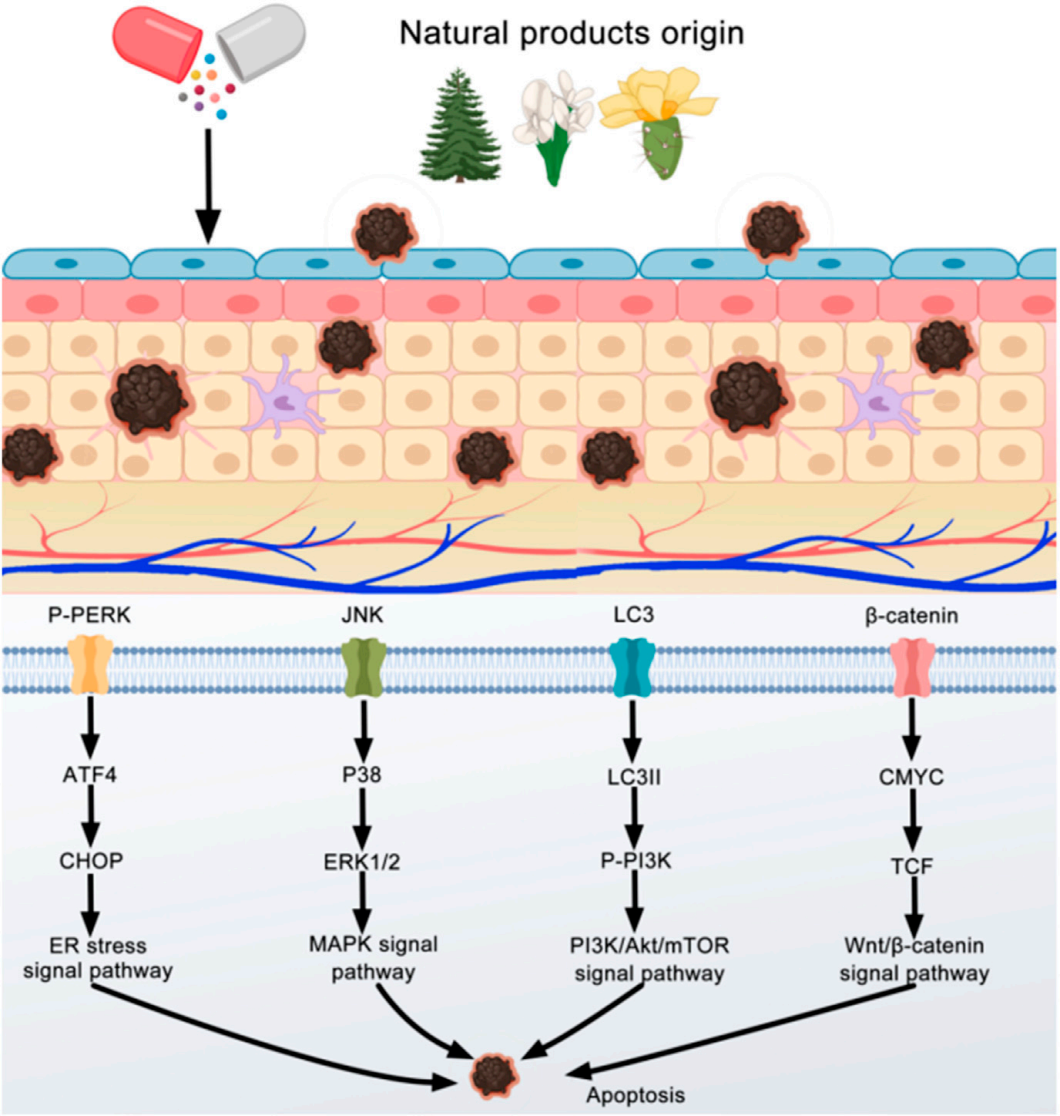
Natural products can induce apoptosis of melanoma cells through various mechanisms.

By screening and summarizing the natural products that can induce melanoma cell apoptosis, we found that many *in vitro* and *in vivo* studies confirmed that natural products could induce melanoma cell apoptosis through various mechanisms. We classify these mechanisms as follows: 1) Wnt/β-Catenin signal pathway, 2) mediated endoplasmic reticulum (ER) stress signal pathway, 3) MAPK signal pathway, 4) phosphoinositol-3-kinase/protein kinase B/mammalian target protein of rapamycin (pl3k/akt/mTOR) signal pathway, and 5) others. As shown in Figure 2.

## 3 Wnt/β-catenin signal pathway

**FIGURE 3:**
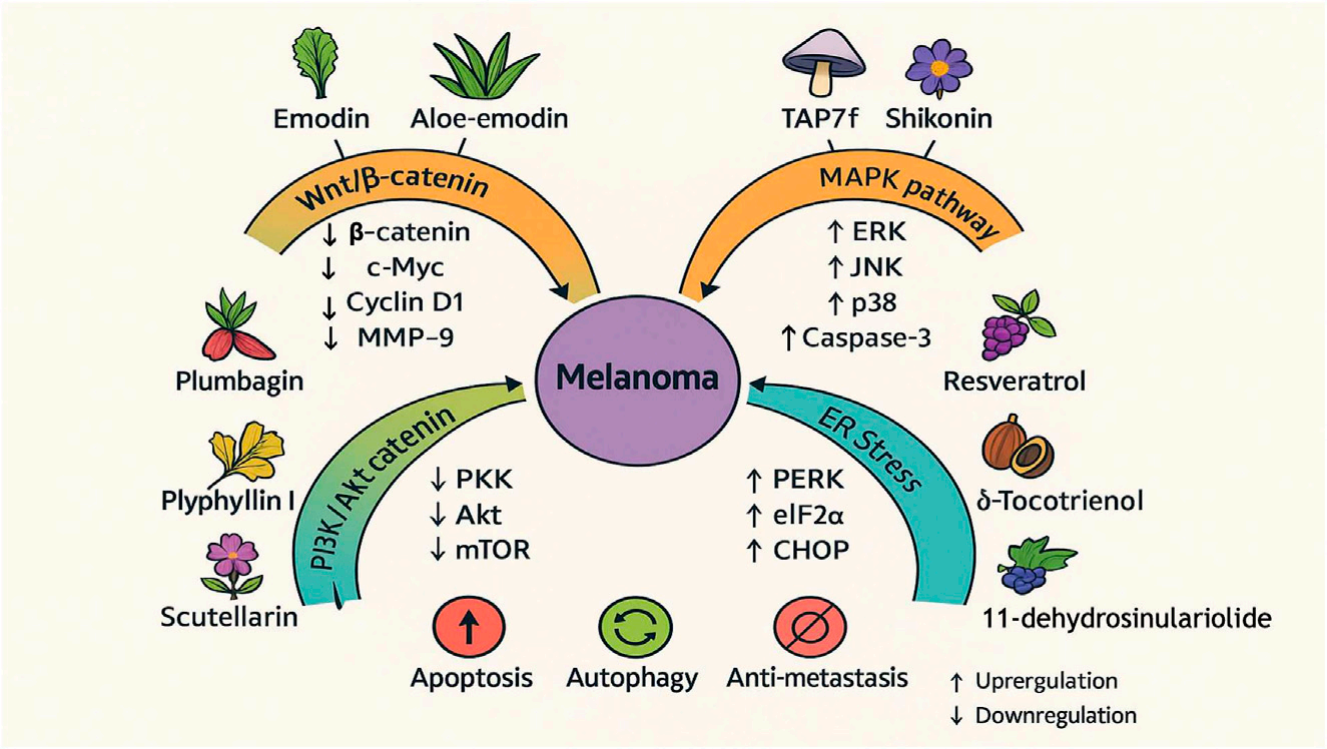
Compounds affect changes in melanoma markers through related mechanisms.

Previous studies have shown that Wnt/β-catenin signaling plays a critical role in embryonic development, cell differentiation and proliferation, and the ability of stem cells to self-renew (Riccardo et al., 2014; Li and Hou, 2018). It has now been demonstrated that the Wnt/β-catenin pathway regulates neural crest melanocyte formation and development and plays a vital role in the pathogenesis of melanoma (Umar et al., 2022; Natarelli et al., 2023). Several specific inhibitors have been developed and have been used in early clinical trials. However, there are insufficient nanotherapeutic activities for further registration trials (Voronkov and Krauss, 2013). Therefore, the Wnt/β-catenin pathway should be a priority molecular target for new drug development to expand the efficacy of clinical immunotherapy, as shown in Figure 3.

### 3.1 Emodin

**FIGURE 4:**
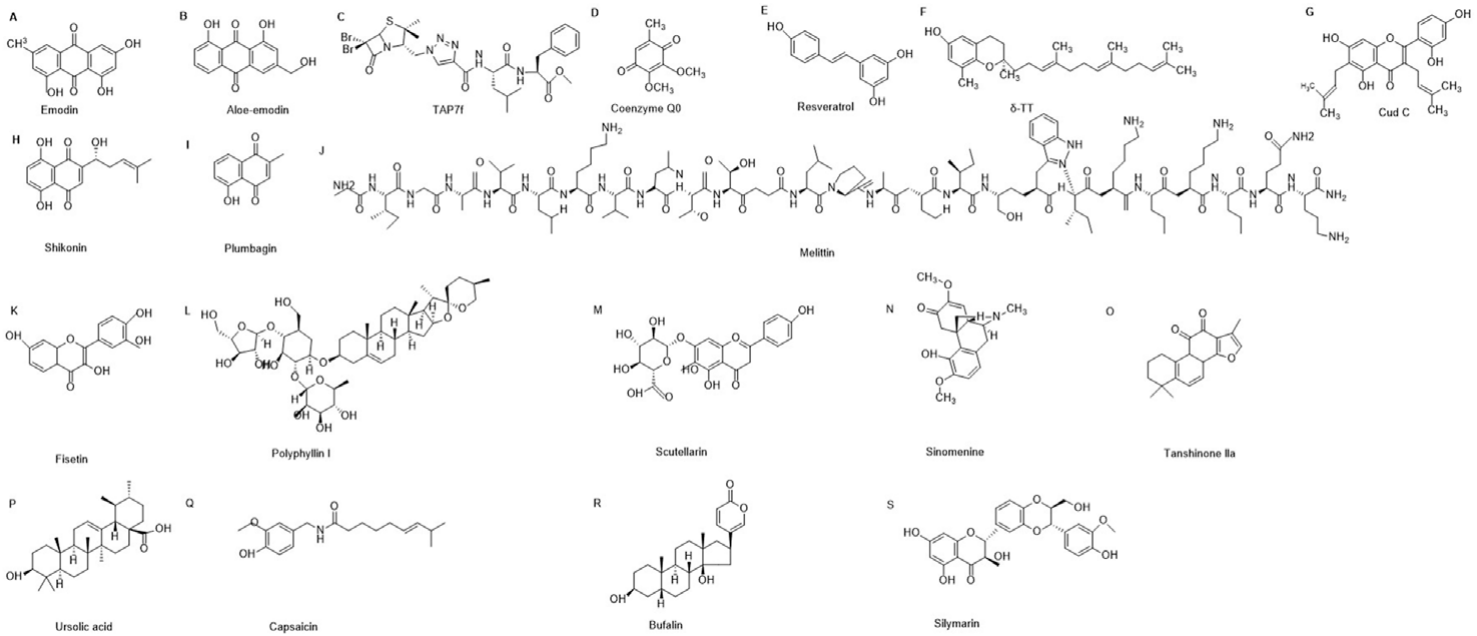
Chemical structures of various compounds labeled **(A–S)**. Some examples include Emodin **(A)**, Aloe-emodin **(B)**, TAP7f **(C)**, Coenzyme Q0 **(D)**, Resveratrol **(E)**, δ-TT **(F)**, Cudraflavone C **(G)**, Shikonin **(H)**, Plumbagin **(I)**, Melittin **(J)**, Fisetin **(K)**, Polyphyllin I **(L)**, Scutellarin **(M)**, Sinomenine **(N)**, Tanshinone IIa **(O)**, Ursolic acid **(P)**, Capsaicin **(Q)**, bufalin **(R)** and Silymarin **(S)**.

Emodin is also known as 1,3,8-trihydroxy-6-methylanthraquinone, its chemical structural formula as shown in Figure 4, and contains the remaining 18 natural products. It is the active ingredient in the root and rhizome of Rhubarb. Studies have shown that emodin has a wide range of pharmacological effects, including antibacterial, immunomodulation, anti-inflammatory, anti-tumor and enhanced cancer chemotherapy (Semwal et al., 2021; Zhang et al., 2022; Wang et al., 2023). It has been suggested that rhodopsin may impede the migration and invasion of melanoma B16F10 and A375 cells by inhibiting the Wnt/β-Catenin signaling pathway (Liu et al., 2021). Furthermore, rhodopsin also inhibits the growth of cancer cells by downregulating CD155 in melanoma B16F10 cells (Fang et al., 2019). Therefore, rhodopsin may be a potential drug for treating highly metastatic melanoma.

### 3.2 Aloe-emodin

Aloe-emodin is an anthraquinone extract from the vine with the chemical formula C15H10O5 (Bhat and Kudva, 2011). Aloe-emodin has numerous pharmacological effects, including anti-inflammatory, immunomodulatory, and wound healing (Dong et al., 2020; Luo et al., 2024). New evidence showed that aloe-emodin exhibited anti-cancer ability in various cancers by inhibiting cell proliferation, migration, and invasion (Sanders et al., 2017). It was experimentally demonstrated that the proliferation, migration, and invasion ability of A375 and SK-MEL-28 melanoma cells treated with aloe-emodin were significantly inhibited, and the growth of A375 and SK-MEL-28 melanoma cells was affected by the inactivation of the Wnt/β-catenin signaling pathway. Furthermore, aloe-emodin also significantly inhibited the growth of A375 and SK-MEL-28 cells in the mouse model of transplantation tumors (Du et al., 2021). Therefore, aloe-emodin could be a potential treatment for melanoma.

### 3.3 TAP7f

TAP7f have anti-proliferative activity against different tumor cell lines (Bellizzi et al., 2022). Its anti-proliferation activity against tumor cells is 30 times higher than that against normal cells. Research has demonstrated that TAP7f exerts antitumor effects by inducing cell cycle arrest and activating death receptors and mitochondria-dependent apoptotic pathways in melanoma B16-F0 cells. Moreover, the melanoma tumor growth in the mouse model treated with TAP7f was reduced by 70% (Blank et al., 2018). New research has recently revealed that TAP7f may inhibit melanoma cell proliferation, migration, and invasion by blocking the Wnt/β-catenin pathway, reducing β-catenin nuclear translocation, and decreasing β-catenin and specific downstream targets (Barrionuevo et al., 2020).

### 3.4 Coenzyme Q0 (CoQ0)

CoQ0 also known as 2,3 dimethoxy-5-methyl-1,4 benzoquinone, is a common ubiquinone compound with redox activity widely found in biological mitochondria. CoQ0 has been demonstrated to have proliferative inhibitory effects on various cancer cell lines (Chung et al., 2014; Wang et al., 2017). *In vitro* experiments have shown that CoQ0 inhibits the proliferation of melanoma B16 cells by suppressing β-catenin-induced transcriptional activation and by nuclear translocation of the β-catenin proteasome. In B16F10 xenograft mice, the expression of β-catenin, Cyclin D1, Survivin, and MMP-9 was significantly reduced in the CoQ0-treated animal model (Hseu et al., 2016). These data demonstrate that CoQ0 inhibits cell growth and apoptosis and prevents metastasis by suppressing melanoma cells’ Wnt/β-linked protein signaling pathway.

## 4 ER pathway

ER is a eukaryotic cell organelle responsible for protein synthesis and calcium (Ca^2+^) signaling (Zheng et al., 2023). Previous studies have shown ER stress is strongly associated with cancer (Oakes, 2017). ER Stress initiates the unfolded protein response (UPR) to re-establish ER homeostasis as an adaptive pathway in cancer (Urra et al., 2013). Several studies have reported the involvement of ER stress in regulating the apoptotic mechanisms leading to melanoma cell death (Selimovic et al., 2013; Wroblewski et al., 2013). Accumulating evidence has shown that ER stress-induced autophagy may be a potential pro-survival mechanism contributing to melanoma development and resistance to BRAF inhibitors (Meng et al., 2015). Recently, there has been research on a novel natural compound called kuwanon H that can induce cytotoxic ER stress, inhibit cell viability, and induce apoptosis in melanoma cells. And it can induce ER stress-induced autophagosome formation through the ATF4-DDIT3-TRIB3-AKT-MTOR axis (Hu et al., 2023). Thus, modulation of ER stress may improve existing cancer therapies and identify new targets for therapeutic intervention in melanoma.

### 4.1 Resveratrol

Resveratrol is a dietary product found in grapes, vegetables, and berries. Resveratrol has been reported to affect each stage of carcinogenesis. Many studies demonstrate that resveratrol can be an ideal anti-cancer molecule because it has a cytotoxic effect on cancer cells (Jang et al., 2022). Studies have shown that resveratrol may induce ROS production and ER stress, thereby hindering the antioxidant effect of resveratrol and enhancing apoptosis of melanoma A375SM cells (Heo et al., 2018), revealing the potential use of resveratrol in the treatment of melanoma. A study has found that resveratrol downregulates the protein level of anti apoptotic protein Bcl-2 and activates Bax by promoting the degradation of Bcl-2 and the release of cytochrome c. In addition, they found that PKM2 plays a crucial role in triggering cell apoptosis. Summarizing that resveratrol in melanoma cells and downregulating the Erk/PKM2/BCl-2 axis seems to be a new method for preventing or treating melanoma (Zhao et al., 2018).

### 4.2 δ-TT

δ-TT is found in a wide variety of natural products. Due to its powerful neuroprotective, anti-inflammatory, antioxidant, and cholesterol-lowering potential (Ahsan et al., 2014; Peh et al., 2016). It works in various chronic diseases, and δ-TT shows antitumor activity as well (Wang et al., 2022). It has been demonstrated that δ-TT triggers cell death and activates ER stress-related pathways such as PERK/p-eIF2α/ATF4/CHOP and IRE1α to induce apoptosis in melanoma cells and that δ-TT significantly inhibits tumor growth rate in A375 melanoma animal model (Montagnani et al., 2016). These studies suggest that δ-TT may also be a potential drug for the treatment of melanoma.

### 4.3 Cudraflavone C (Cud C)

Cudraflavone C is a natural flavonoid compound with anti proliferative activity, which was initially found to inhibit melanin production (Zheng et al., 2012; Chan et al., 2021). Tumor specific apoptosis of rectal cancer cells can be induced by targeting the PI3K/AKT pathway (Soo et al., 2018). Research proves that cudraflavone C induces apoptosis in A375.S2 melanoma cells by increasing the production of mitochondrial ROS, activating p38, ERK and JNK, and increasing the expression of apoptotic proteins (Lee et al., 2017). Thus, cudraflavone C may be considered a potential therapeutic agent for treating malignant melanoma.

### 4.4 11-dehydrosinulariolide

11-Dehydrosinulariolide is a cembranolide analog, with a variety of biological activities (Chen et al., 2016). It has also been shown that 11-dehydrosinulariolide induces apoptosis in human melanoma cells by up-regulating PERK/eIF2α/ATF4/CHOP and ATF6/CHOP coupled with elevated ER stress chaperones GRP78, GRP94, calcium-linked protein, calreticulin, and PDI, which impede cystein-dependent mitochondrial function and ER stress pathway in human melanoma cells A2058 (Su et al., 2012). The present findings suggest that 11-dehydrosinulariolide is an effective compound against powerful melanoma cells *in vitro*, facilitating the drug development of anti-melanoma drugs.

## 5 MAPK

The MAPK cascade regulates cell proliferation, growth, and migration, an ordinarily active pathway but overactivated in almost all melanomas (Long et al., 2014). Mutations in NRAS or BRAF genes were observed in 80% of melanoma or melanocytic nevus cases, confirming the critical role of the MAPK pathway (Huang et al., 2023). New drugs targeting the MAPK pathway have produced excellent clinical responses in melanoma treatment, from the discovery of BRAF mutations in melanoma in 2002 to the FDA’s approval of the first BRAF inhibitor, vemurafenib, for melanoma treatment in 2011, therapies targeting the MAPK pathway have proven effective in less than a decade (Cheng et al., 2013). However, sequential treatment with BRAF/MEK inhibition and immunotherapy may increase the toxicity of sepsis-like syndrome and is associated with severe side effects (Moreira et al., 2021). Consequently, there is a need to develop new drugs that target the MAPK pathway for the treatment of melanoma.

### 5.1 Shikonin

Shikonin is a natural product isolated from comfrey, a member of the comfrey family, and has long been used in China to treat inflammation, burns, ulcers, infections, and cancer (Sun et al., 2022). Experiments have shown that increased phospho-ERK1/2, phospho-JNK, and phospho-p38 were observed in phycocyanin-treated cells, and total-ERK1/2, total-JNK, and total-p38 tended to decrease in phycocyanin-treated cells. Identifying apoptosis in A375SM melanoma cells induced by comfrey treatment through MAPK pathway. In a mouse animal model, the tumor volume was reduced by Shikonin administration. P38, an essential protein in the MAPK pathway, was significantly increased (Liu et al., 2019). The above results demonstrate that the apoptosis induction of paclitaxel in A375SM melanoma cells seems to be mediated by the expression of ERK and JNK proteins in the MAPK pathway, especially the expression of p38 (Lee et al., 2021).

### 5.2 L. barbarum extracts

L. barbarum is a traditional Asian food and medicine rich in zeaxanthin. Many studies have shown that L. barbarum extract has immunomodulatory effects and anti-tumor activity (Ceccarini et al., 2016; Wawruszak et al., 2016). L. barbarum extract containing zeaxanthin has been shown to inhibit the proliferation of human melanoma A375 cells, induce the expression of MAPK factors ERK1/2, JNK, and p38 in A375 skin cells, upregulate total NF-kB, thereby inducing apoptosis and reducing tumor cell growth. Therefore, L. barbarum extract may be used as an adjuvant for standard antitumor chemotherapy (Cenariu et al., 2021).

### 5.3 Plumbagin (PLB)

PLB is a naphthoquinone derivative derived mainly from plants that exhibit anti-cancer potential in different cancers (Yin et al., 2020; Zhang et al., 2020). Plumbagin was shown to downregulate MAPK-related genes, including Map3k3, MAPK14, Braf, c-Myc, and MAPK1, and induce enhanced gene expression of Igfbp5 and Pten, thus promoting further activation of the MAPK pathway, and thus acting as an anti-invasive and anti-metastatic agent in melanoma B16F10 cells (Alem et al., 2020). Meanwhile, PLB can trigger cell-specific cytotoxic effects and metabolic responses in melanoma A375 cells (Zhang et al., 2021). Combining Celecoxib and Plumbagin reduces the proliferation of melanoma cells and inhibits COX-2 and STAT3-mediated tumor vascular development, thereby inducing apoptosis in human fibroblast FF2441 cells (Gowda et al., 2017). Hence, it is necessary to investigate PLB as an antitumor agent and further develop its potential clinical applications.

### 5.4 Melittin

Bee Venom (BV) is a natural toxin produced by honey bees (*Apis mellifera*) and has been widely used as a traditional medicine for many diseases (Sadek et al., 2024). Melittin is the main bioactive component of BV. Moreover, in the current study, melittin has been shown to act against melanoma A375 cells by downregulating PI3K/AKT/mTOR and MAPK signaling pathways. Moreover, the growth, migration, and invasion of melanoma B16F10, A375SM, and SK-MEL-28 cells treated with different melittin concentrations were inhibited (Lim et al., 2019). Ultrasmall lipid nanoparticles driven by mixed melittin proteolytic peptides inhibit tumor cell growth in a mouse melanoma model, with no side effects observed (Huang et al., 2013). The current findings suggest that melittin could be used as a potential targeting agent in melanoma treatment.

### 5.5 Fisetin

Fisetin (3,3′,4′,7-tetrahydroxyflavone) is a dietary flavonoid found in various fruits and vegetables, including strawberries, grapes, apples, onions, and cucumbers (Khan et al., 2018). In many biological activities, including neuroprotective, anti-arthritic, and anti-allergic activities (Ahmad et al., 2017; Zheng et al., 2017). Fisetin was shown to inhibit phosphorylation of MEK1/2 and ERK1/2 in the MAPK signaling pathway activation pathway and inhibit NFκB in signaling to reduce melanoma A375 cell invasion and metastasis. Fisetin reduced the invasion of melanoma cells into the dermis in 3D skin consisting of A375 cells mixed with average human keratin-forming cells embedded in a collagen-constricted fibroblast matrix (Pal et al., 2014). In 3-D melanoma constructs, fisetin inhibits the growth of human melanoma A375 cells by directly binding to p70S6K and mTOR (Syed et al., 2014). The above experimental results demonstrate that fexofenadine can be developed as a potential anti-melanoma drug.

## 6 PI3K/AKT/mTOR

PI3K/Akt/mTOR pathway regulates cell proliferation, growth, size, metabolism, and motility (Alzahrani, 2019). Since multiple genes of the PI3K/Akt/mTOR signaling pathway are frequently altered in human cancers, dysregulation through mutation or amplification of genes involved in the PI3K pathway, loss of the tumor suppressor PTEN or over-activation of RTK leads to tumor progression and metastasis. The genes comprising this pathway are an essential molecular therapeutic target for human cancers (Murugan et al., 2013; Arafeh and Samuels, 2019).

### 6.1 Polyphyllin I (PPI)

PPI is a bioactive component derived from Paris polyphylla with a wide range of biological and pharmacological activities (Yang et al., 2024; Zhang et al., 2024). It was demonstrated that the expression of p-PI3K, p-Akt, and p-mTOR was significantly reduced in A375 cells after PPI treatment, and inhibited melanoma cell proliferation and enhanced melanoma cell apoptosis by suppressing PI3K/Akt/mTOR signaling pathway, which blocked melanoma cells at G0/G1 stage, thus reducing melanoma progression. In addition, the weight and size of melanoma mice treated with PPI was significantly reduced, and apoptosis of melanoma cells was significantly enhanced (Long and Pi, 2020). Therefore, PPI may be a promising, targeted drug for melanoma treatment.

### 6.2 Scutellarin

Scutellarin (4′,5,6-hydroxyl-flavone-7-glucuronide) is a flavonoid derived from Calendula officinalis with a variety of functions including antioxidant, anti-inflammatory, cardioprotective, and vasodilator (Wang and Ma, 2018). Scutellarin promotes A375 cell apoptosis by upregulating bax and cleaved caspase-3 levels, downregulating bcl-2 levels, Inhibit the growth of A375 cells in G0/G1 phase, Enhance cellular autophagy by regulating the levels of Beclin 1, LC3II, and p62. And block the melanoma cell cycle by inhibiting the PI3K/Akt/mTOR signaling pathwa (Li et al., 2019). It has also been demonstrated that lanosterol inhibits the proliferation of human melanoma cells RPMI7951 melanoma cells by targeting TOPK. In addition, *in vivo* experiments demonstrated that SCU inhibited the growth of RPMI7951 cell xenografts and reduced the phosphorylation levels of ERK 1/2 and histone H3 *in vivo* (Mu et al., 2021). It suggests that scutellarin may be a potential compound for treating malignant melanoma.

### 6.3 Bornyl *cis -4-hydroxycinnamate*

Bornyl cis-4-hydroxycinnamate, an active compound isolated from Piper betle stem, could inhibit cell viability, migration, and invasion of A2058 and A375 melanoma cells in a dose-dependent manner. The expression of FAK/PI3K/Akt/mTOR signaling-related proteins, including Akt, p-Akt, PI3K, p-PI3K, mTOR, p-mTOR, and FAK, was reduced in cis-4-hydroxycinnamate treated melanoma cells, which inhibited the metastasis of human melanoma cells through the FAK/PI3K/Akt/mTOR signaling pathway (Yang et al., 2018a). Additionally, cis-4- hydroxycinnamate has been shown to mediate apoptosis in melanoma cells by activating the cystathionine cascade, inducing mitochondrial dysfunction, and causing endoplasmic reticulum stress-related stress mechanisms (Yang et al., 2018b). The above results demonstrate that bornyl cis-4-hydroxycinnamate can potentially be a chemotherapeutic agent for human melanoma development.

### 6.4 Sinomenine

Sinomenine (7,8-didehydro-4-hydroxy-3,7-dimethoxy-17-methylmorphinane-6-one; SIN) is the active compound of the Chinese herb Cyanidin. SIN has an antitumor effect (Gao et al., 2019), including lung cancer and breast cancer (Song et al., 2015; Jiang et al., 2016). Recent studies have shown that SIN inhibits proliferation and promotes apoptosis in melanoma B16F10 cells via PI3K/Akt/mTOR-dependent autophagic pathway. In a melanoma xenograft mouse model, tumor volume and weight were significantly reduced after SIN treatment, and the expression levels of Ki67 and PCNA were significantly reduced (Sun et al., 2018). It suggests that SIN can reduce the tumor growth of melanoma *in vivo*. However, SIN also has some disadvantages, for example, poor gastrointestinal response, short biological half-life, and unstable physicochemical properties (Zhang et al., 2018). Therefore, the role of SIN in the treatment of melanoma deserves further investigation.

### 6.5 Tanshinone IIa (TanIIA)

TanIIA, a compound isolated from Salvia miltiorrhiza, has various biological activities, including apoptosis and autophagy, anti-inflammation, oxidation, anti-thrombosis, and anti-proliferation of vascular smooth muscle cells (Luo et al., 2015; Naz et al., 2020). It was shown that the phosphorylation levels of PI3K, P-AKT, P-mTOR, and P-p7036k1 were reduced in melanoma A375, MV3, and M14 cells treated with TanIIA, which inhibited the proliferation and invasion, and migration ability of melanoma cells, and promoted the autophagosome production of A375, In addition, TanIIA inhibits the development of A375 melanoma-induced tumor weight and volume in mice (Li et al., 2017). Research has found that TanIIA photosensitization has significant toxicity to choroidal melanoma cells and can effectively induce cell apoptosis and necrosis. Increase intracellular ROS levels, decrease mitochondrial membrane potential, and cause cell arrest in G2/M phase (Juan et al., 2021). In a recent report, the first study to link Tan IIA induced ferroptosis with the STAT1/PTGS2 axis in melanoma was found. Tan IIA regulates the key marker of ferroptosis, PTGS2, and knocking down PTGS2 weakens Tan IIA induced ferroptosis in melanoma cells. In addition, they found that Tan IIA stimulated the downregulation of signal transduction and transcription factor STAT1, leading to the downregulation of PTGS2 and inhibiting ferroptosis in melanoma (Chen et al., 2025).

## 7 Other mitochondrial mechanisms of resistance to melanoma

### 7.1 Ursolic acid (UA)

UA3-(β-hydroxy-urs-12-en-28-oic acid) is a pentacyclic triterpenoid compound commonly found in fruits, foods, and medicinal plants (Castro et al., 2015; Huang et al., 2015; Yin et al., 2018). Recent experiments have shown that ursolic acid activates the proteolytic processing of caspase-3 in isolated human melanoma cells and induces apoptotic cell death (Mahmoudi et al., 2015; Alvarado et al., 2018). Nanoemulsion of ursolic acid isolated from egg flowers enhances melanoma cells’ antioxidant and cytotoxic activity. When UA and chloroquine were synergistically applied to B16F10 mouse melanoma and A375 human melanoma cells, cell viability was strongly reduced (Junco et al., 2015). The above results suggest that ursolic acid could be a new potential tool for further research as an anti-cancer agent.

### 7.2 Capsaicin

Capsaicin is a component of chili peppers, the active ingredient in chili peppers. Capsicum is widely used as a pungent spice in food. Previous studies have demonstrated that capsaicin has anti-inflammatory, analgesic, anesthetic, and detoxifying effects (Bernstein et al., 2011; Patel et al., 2014; Fattori et al., 2016). Capsaicin was shown to affect cancer cell viability negatively and induce apoptosis in human melanoma A375 and C8161 cells by activating cleaved caspase-3 and PARP (Chu et al., 2019). There is also evidence that capsaicin inhibits the growth of SK-MEL-28 melanoma cells and increases apoptosis by inhibiting plasma membrane NADH oxidase activity (Morré et al., 1996). Capsaicin also inhibits the migration of B16-F10 melanoma cells by suppressing the PI3K/AKT/Rac-1 pathway (Shin et al., 2008). Therefore, capsaicin administration can be an effective method for the treatment of malignant melanoma.

### 7.3 Toad venom

Toad venom is a traditional natural medicine that has been used for centuries in China, and a growing body of research suggests that toad venom is a source of lead compounds for the development of potential cancer therapeutics (Li et al., 2021). Na^+^/K^+^ATPase is overexpressed in a variety of cancer types, including metastatic melanoma. Recent studies have shown that toad venom’s active ingredient, toadienolactone, acts on the Na^+^/K^+^-ATPase pump to exert anti-proliferative effects on melanoma cells (Soumoy et al., 2020). Bufalin, another active ingredient, can act through extrinsic and mitochondrial-mediated signaling pathways to trigger apoptosis (Hsiao et al., 2012). Bufalin can also potentially stimulate tyrosinase activity to promote melanin synthesis (Zhang et al., 1992). It may lead to the production of toxic melanin precursors, which inhibit melanoma growth.

### 7.4 Silymarin

Silymarin is the main bioactive component of silybum marianum and has long been used for the prevention of allergies and liver damag (Gillessen and Schmidt, 2020). Several studies have demonstrated the chemopreventive or chemotherapeutic effects of silymarin on various cancers (Dagne et al., 2011; Ramasamy et al., 2011). It has been shown that silymarin induces cell cycle arrest and inhibits the growth of human melanoma SK-MEL-5 and SK-MEL-28 cells in the G1 phase by blocking MEK1/2-RSK2 signaling, leading to a decrease in the activation of various transcriptional regulators of proliferation genes in melanoma, such as nuclear factor-kappaB, activator protein-1, and signal transduction and transcriptional activator 3. Silymarin also attenuates the growth of melanoma xenografts in nude mice (Lee et al., 2013). Furthermore, the combination of cold atmospheric plasma and silymarin nanoemulsion inhibited the HGF/c-MET signaling pathway to promote apoptosis in G-361 human melanoma cells and reduce tumor growth in a tumor xenograft nude mouse model (Adhikari et al., 2019), as shown in Table 1.

**TABLE 1:** Natural products have been proven to have an antagonistic effect on melanoma through *in vitro* and *in vivo* experiments.

Signal pathway	Natural products	Cell/animal model	Mode of action	Concentration	Ref.
Wnt/β-catenin signal pathway	Emodin	B16F10	MMP-2↓ MMP-9↓	20 μm 40 μm 60 μm	(Liu et al., 2021)
		A375			
	Aloe-emodin	SK-MEL-28 A375	caspase −3↑ bax ↑ cyclinD1↓ c-myc↓ bcl-2↓ wnt3a↓ β-catenin↓ p-GSK3β↓	5 μg/mL 10 μg/mL 15 μg/mL	(Du et al., 2021)
		nude mouse model	the weight of the tumors↓	20 μg/mL	
	TAP7f	B16F10	cyclin-D1↓ c-Myc↓ αvβ3↓	5 μm 10 μm 15 μm 20 μm	(Blank et al., 2018; Barrionuevo et al., 2020)
		A375	cyclin-D1↓ c-Myc↓ e-cadherin↑	5 μm 10 μm 15 μm 20 μm	
	Coenzyme Q_0_	B16F10	GSK3β↓ c-Myc↓ cyclin D↓ survivin↓ MMP-2/9↓ TIMP-1/2↑	5 μm 10 μm 15 μm 20 μm	(Hseu et al., 2016)
Mediated ER stress signal pathway	Resveratrol	A375SM	ROS↑ p-eIF2α↑ CHOP↑	1 μm 10 μm	(Heo et al., 2018)
		MV3	Bcl-2↓ ERK1/2 ↓ PKM2↓	50 μm 100 μm 200 μm	(Zhao et al., 2018)
	δ-TT	A375	PERK↑ IRE1α↑ ERO1α↑	5 μm 10 μm 15 μm 20 μm	(Montagnani et al., 2016)
	Cudraflavone C	A375	ROS↑ caspase-3/7/9↑ puma↑ bax↑ bad↑ bid↑ apaf-1↑	9.2 μm	(Lee et al., 2017)
	11-dehydrosinulariolide	A2058	PERK↑ eIF2α↑ ATF4↑ CHOP↑	5 μm 10 μm 15 μm 20 μm	(Su et al., 2012)
MAPK signal pathway	Shikonin	A375	ERK1/2↑ JNK↑ P38↑ ROS↑	1 μm 2 μm 4 μm 8 μm	(Liu et al., 2019)
		nude mice model	inhibits the growth and progression	4 mg/kg	(Lee et al., 2021)
	*L. barbarum* Extracts	A375	ERK↑ JNK↑ p38↑ NF-kB↑	100 μm 200 μm	(Cenariu et al., 2021)
	Plumbagin	B16F10	map3k3↓ mapk14↓ braf↓ c-Myc↓ mapk1↓ Igfbp5↑ pten↑	1 μ/m 2 μ/m	Alem et al. (2020)
		A375		1 μm 3 μm	(Zhang et al., 2021)
	Melittin	B16F10	PI3K↓ AKT↓ mTOR↓ MAPKs↓	0.5 μg/mL 1 μg/mL 1.5 μg/mL	(Lim et al., 2019)
	Fisetin	A375	MEK1/2↓ ERK1/2↓ NF-κB↓	5 μm 10 μm 20 μm	(Pal et al., 2014)
		SK-MEL-28		5 μm 10 μm 20 μm	
PI3K/AKT/mTOR signal pathway	Polyphyllin I	A375	p-PI3K↓ p-Akt ↓ p-mTOR↓	1.5 mg/L 3 mg/L 6 mg/L	(Long and Pi, 2020)
		BALB/c nude mice	Inhibit melanoma growth	1.5 mg/L 3 mg/L 6 mg/L	
	Scutellarin	A375	p-Akt↓ p-mTOR↓ VEGF-A↓ MMP-2↓ MMP-9↓	5 μm 10 μm 20 μm	(Li et al., 2019)
	Bornyl cis -4-hydroxycinnamate	A2058	akt↓ PI3K ↓ mTOR↓ FAK↓	3 μm 6 μm 9 μm 12 μm 15 μm 18 μm	(Yang et al., 2018a; Yang et al., 2018b)
		A375			
	Sinomenine	B16F10	eclin1↑ bcl-2↓ bax↑ caspase-3↑	12.5 μm 25 μm 50 μm 100 μm	(Sun et al., 2018)
		BALB/c mice	reduce the tumor growth	20 μm	
	Tanshinone IIa	A375	PI3K↓ P-AKT↓ P-mTOR↓ P-p7036k1↓	0.5 μg/mL 1 μg/mL 2 μg/mL 4 μg/m	(Li et al., 2017)
		MV3			
		M14			
		BALB/e nude mice	reduce the tumor growth	1 μg/mL 2 μg/mL 4 μg/m	

Abbreviations: ROS, reactive oxygen species; CHOP, C/EBP, homologous protein; p-eIF2α, phosphorylated eukaryotic initiation factor 2α; Bcl-2, b-cell lymphoma-2; Wnt3a, recombinant wingless type MMTV, integration site family, member 3A; GSK-3β,glycogen synthase kinase 3β; PKM2, M2-type pyruvate kinase; PERK, protein kinase PKR-like ER, kinase; IRE1α, inositol-requiring enzyme 1α; MMP, matrixmetalloproteinase; TIMP, tissue inhibitor of metalloproteinases; NF-kB, nuclear factor kappa-B; mTOR, mammalian target of rapamycin; Igfbp5, insulin-like growth factor-binding protein 5; ATF4,recombinant activating transcription factor 4; Apaf-1, apoptotic protease activating factor 1.

## 8 Discussion

Currently, the etiology of melanoma is generally believed to involve: 1) exposure to sunlight, 2) racial and genetic factors, 3) malignant transformation of benign pigmented nevi, 4) trauma and chronic irritants, which are also considered to be associated with melanoma development. Dacarbazine (1975), high-dose interleukin-2 (1992), and high-dose interferon α-2b (1995) were approved by the U.S. Food and Drug Administration (FDA) for the treatment of advanced melanoma. Although these advancements over 2 decades have offered hope for treating melanoma, the overall therapeutic outcomes remain unsatisfactory (Atkins et al., 1997; Kirkwood et al., 2004; Yang and Chapman, 2009). Since the early 2000s, the emergence of targeted therapies has highlighted the critical role of oncogene mutations—particularly in BRAF and NRAS—in activating cellular survival and proliferation pathways, including the MAPK and PI3K/AKT signaling cascades. The inhibition of mutated BRAF proteins by selective kinase inhibitors represents the most significant breakthrough in targeted melanoma therapy to date (Lim et al., 2017). Among the most established therapies targeting the BRAFV600E mutation site are vemurafenib (approved in 2011) and dabrafenib (approved in 2013), which serve as first-line treatments for advanced BRAF-mutant melanoma (Garbe and Eigentler, 2018; Long et al., 2024). Extensive studies have demonstrated that the immunosuppressive molecular marker PD-L1 plays a pivotal role in the mechanism of tumor immune evasion. Monoclonal antibodies such as nivolumab and pembrolizumab, which target PD-1, and atezolizumab, which targets PD-L1, have been shown to effectively block the PD-1/PD-L1 interaction (Hamanishi et al., 2016). The immune checkpoints they mediate inhibit the activity of reactivated immune cells, thereby enabling tumor cells to escape immune surveillance. Moreover, nivolumab and pembrolizumab have been approved as first-line therapies for surgically unresectable or metastatic malignant melanoma. Targeted therapy and immunotherapy have significantly altered the treatment landscape for both resectable and unresectable melanoma, representing a major advance in melanoma management.

Although progress has been made in melanoma treatment, immunosuppressive agents currently employed in clinical practice are characterized by limited specificity, and their use is constrained by toxic side effects and elevated costs. To date, the clinical efficacy of monotherapy remains insufficiently defined, and the development of individualized treatment regimens and combination approaches based on a patient’s immune status remains a priority for future investigation. Consequently, the exploration of novel natural products and their extracts as integral components of combination therapy has been widely investigated in oncology. These compounds are characterized by low toxicity, wide availability, and modest cost (Sflakidou et al., 2022). A comprehensive understanding of the relationship between chemical structure and biological activity, along with the optimization of structure–activity relationships, can help clarify the diverse structures of plant-derived compounds and facilitate the investigation of correlations between nanostructures and biological activity. This, in turn, may enhance the immunological potential of compound extracts and support the rational design of more effective drug candidates. In addition, mouse models represent the most widely utilized animal models in preclinical research. Melanoma mouse models are typically categorized into three types: inducible models (e.g., UV-induced models and chemically induced models using DMBA or TPA), transplantable models (including syngeneic and xenograft models), and transgenic models (such as Lum−/− knockout mice, TyrN-RasQ61K transgenic mice, and BrafV600E transgenic mice) (Kuzu et al., 2015; Day et al., 2017), these models have become essential tools for investigating the pathogenesis, metastasis, and therapeutic evaluation of melanoma. The models discussed in this review are broadly classified into two categories: *in vivo* and *in vitro* experimental systems. *In vitro* studies primarily involve melanoma cell lines such as B16F10, A375, SK-MEL-28, and A2058, whereas *in vivo* studies utilize animal models including BALB/c nude mice. In addition, other animal models such as C57BL/6, C3H/HeN, and CB17-SCID mice have also been utilized in various melanoma-related studies (Liu et al., 2018; Saleh, 2018). This research aims to identify susceptibility and risk factors for melanoma development, assess the efficacy of immunotherapies and tumor–immune cell interactions, elucidate the signaling pathways involved in tumor progression, clarify gene functions, and define potential therapeutic targets.

Natural product–derived compounds have been applied in clinical oncology; however, their clinical utility is often limited by inherent physicochemical and pharmacokinetic properties. For instance, poor water solubility, limited biocompatibility, low oral bioavailability, chemical instability, and suboptimal pharmacokinetics significantly hinder their clinical translation (Bitterman and Chung, 2015; Khoury et al., 2015; Cai et al., 2016; Kharat et al., 2017; Wen et al., 2017; Velavan et al., 2018; Wei et al., 2019). Therefore, there is a growing need for further studies to clarify the bioavailability and pharmacokinetic behavior of these bioactive compounds. Natural products in melanoma therapy are currently in the early stages of investigation, and their anticancer effects appear to vary in a dose-dependent manner. Although physiological effects have been observed in animal models, there are currently few clinical studies assessing the efficacy of these compounds in humans.

However, these active ingredients exhibit low bioavailability, poor stability, and limited water solubility, which restrict their clinical application and highlight the need for new strategies to enhance their absorption and therapeutic efficacy. In particular, the application and development of nanotechnology has emerged as a promising approach. In conclusion, natural product–derived compounds represent promising anticancer agents that warrant further investigation to better elucidate the mechanisms underlying their antitumor activity. Moreover, combining these compounds with conventional antitumor therapies may enhance their therapeutic potential through synergistic effects and reduced toxicity. Such combinations may offer safer and more effective strategies for clinical intervention. Natural products are expected to contribute to future breakthroughs in tumor immunotherapy.

Overall, the treatment of melanoma continues to face numerous challenges, including: 1) significant biological differences between animal and cell-based models and human physiology, such as variations in genome organization, gene regulation, cell types, and organ structures, which limit the ability to fully replicate human pathological processes; 2) uncertainty regarding how newly identified compounds can achieve optimal bioactivity *in vivo* to exert therapeutic effects; 3) incomplete understanding of the mechanisms of action of novel compounds; 4) the potential for immune-related toxicities associated with the widespread use of bioactive agents; 5) unclear interactions between compound-based therapies and existing targeted or immunosuppressive treatments; and 6) limitations related to sample size and study duration in experimental research. Therefore, in addition to fully understanding the advantages, disadvantages, and applicability of these compounds and animal models, it is also necessary to develop individualized diagnosis and treatment plans for different types of melanoma to provide valuable information for optimizing melanoma models and drug evaluation. Furthermore, the application of drug-specific biomarkers may enable more precise and personalized treatment approaches. The research and development of new drugs remain a long-term task that requires joint efforts from researchers.

## Abbreviations

PI3K, phosphoinositol-3-kinase; PDL-1, programmed death ligand 1; CDKN, cyclin dependent kinase inhibitors; ERK, extracellular signal-regulated kinase; Akt, protein kinase B; mTOR, mammalian target protein of rapamycin; TAP7f, triazolylpeptidyl penicillin derivative; JNK, c-Jun N-terminal kinase; δ-TT, vitamin E delta-tocotrienol; MAPK, mitogen-activated protein kinase; TanIIA, tanshinone IIa; ER, endoplasmic reticulum; UA, ursolic acid; CoQ0, coenzyme Q0; UPR, unfolded protein response; Cud C, cudraflavone C; PLB, plumbagin; BV, bee venom; PPI, polyphyllin I. programmed cell death ligand; PD-L1; programmed cell death, PD-1.
